# Risk factors for cutaneous reactions to allopurinol in Kinh Vietnamese: results from a case-control study

**DOI:** 10.1186/s13075-020-02273-1

**Published:** 2020-08-03

**Authors:** Minh Duc Do, Thao Phuong Mai, Anh Duy Do, Quang Dinh Nguyen, Nghia Hieu Le, Linh Gia Hoang Le, Vu Anh Hoang, Anh Ngoc Le, Hung Quoc Le, Pascal Richette, Matthieu Resche-Rigon, Thomas Bardin

**Affiliations:** 1 Center for Molecular Biomedicine, University of Medicine and Pharmacy, at Ho Chi Minh City, Vietnam; 2 Department of Physiology, Pathophysiology and Immunology, Faculty of Medicine, University of Medicine and Pharmacy, Ho Chi Minh City, Vietnam; 3 Department of Physiology, Pathophysiology and Immunology, Pham Ngoc Thach University of Medicine, Ho Chi Minh City, Vietnam; 4 French Vietnamese Research Center on Gout and Chronic Diseases, Vien Gut Medical Centre, Ho Chi Minh City, Vietnam; 5 Department of Scientific Research, Cho Ray Hospital, Ho Chi Minh City, Vietnam; 6 Department of Tropical Disease, Cho Ray Hospital, Ho Chi Minh City, Vietnam; 7 Université de Paris, U1132, INSERM, 75010 Paris, France; 8 Department of Rheumatology, AP-HP, Lariboisière hospital, 2 rue A. Paré, 75010 Paris, France; 9 Université de Paris, ECSTRRA Team U1153, INSERM, 75010 Paris, France; 10 Department of Biostatistics, AP-HP, Saint-Louis hospital, 75010 Paris, France

**Keywords:** Gout, Allopurinol, Skin reactions, Kinh Vietnamese, Risk factors, HLA-B*58:01

## Abstract

**Objective:**

The aim of this study was to investigate risk factors for cutaneous adverse reactions (CARs) in Kinh Vietnamese.

**Methods:**

All patients were prospectively recruited in Ho Chi Minh City. Presence of the HLA-B*58:01 allele was determined by real-time PCR-sequence-specific amplification by using the PG5801 Detection Kit (Pharmigene, Taipei). Patients with severe (SCARs) and mild (MCARs) CARs and controls were compared for differences in features prospectively collected, and odds ratios (ORs) with 95% confidence intervals (CIs) were estimated.

**Results:**

On comparing 32 patients with SCARs and 395 tolerant controls, we identified eight strong risk factors: increased age (OR 15.1 [95% CI 5.8–40.1], *P* < 0.0001), female sex (OR 333 [40–43,453], *P* < 0.0001), allopurinol for asymptomatic hyperuricemia (OR 955 [120–125,847], *P* < 0.0001), allopurinol starting dose > 150 mg (OR 316 [101–122], *P* < 0.0001), diuretics intake (OR 304 [35–40,018], *P* < 0.0001), eGFR < 60 ml/min/1.73 m^2^ (OR 100 [32–353], *P* < 0.0001), history of allopurinol-induced skin reaction (OR 78 [6–10,808], *P* = 0.004), and HLA-B*58:01 carriage (OR 147 [45–746], *P* < 0.0001). HLA-B*58:01 allele frequency in controls was 7.3%. For MCARs (*n* = 74), risk factors were eGFR < 60 ml/min/1.73 m^2^ (OR 4.9 [1.61–14.6], *P* = 0.006), history of allopurinol-induced skin reaction (OR 27 [2–3777], *P* = 0.01), and asymptomatic hyperuricemia (OR 27 [2–3777], *P* = 0.01).

**Conclusion:**

This study confirmed 8 risk factors, including HLA-B*58:01, for SCARs and identified 3 risk factors for MCARs in Kinh Vietnamese. HLA-B*58:01 genotyping could guide the indication for allopurinol in Kinh Vietnamese patients with gout.

## Introduction

After more than 60 years of discovery and medical use, allopurinol remains the most commonly used urate-lowering agent worldwide, and the first choice of urate-lowering therapy in an overwhelming majority of gout patients [1, 2]. Allopurinol is well tolerated in most cases, but cutaneous adverse reactions (CARs) may occur a few months after allopurinol initiation [3–6]. Mild CARs (MCARs) are the most common. Their incidence has been estimated at 2 to 4% in Caucasian allopurinol initiators [7, 8] and more than 15% in Taiwanese [9]. MCARs feature sparse maco-papular rash and no other organ involvement and quick resolution after drug discontinuation. In contrast, severe CARs (SCARs), characterized by widespread skin lesions, fever, organ involvement, and a mortality rate of 10 to 32% [3, 10, 11], are rare, although their frequency seems increased in Asians and some other non-Caucasian ethnicities [3, 9, 12, 13]. Allopurinol-induced SCARs include Stevens-Johnson syndrome/toxic epidermal necrosis (SJS/TEN), drug reaction with eosinophilia and systemic symptoms (DRESS), and acute generalized exanthematous pustulosis. Allopurinol was identified as the leading cause of SCARs [5, 14] and the second cause of DRESS [15], whereas acute generalized exanthematous pustulosis seems extremely rare.

The risk of hospitalized allopurinol-induced SCARs has been recently estimated at 0.051% (95% CI 0.045–0.059) in US whites and Hispanics, 0.070% (0.039 to 0.115) in Asians, and 0.082% (0.066 to 0.10) in Blacks [13]. Previous studies found that the incidence of SCARs due to allopurinol was higher in Asian than Caucasian populations. The incidence was estimated at 0.16% per year in Thailand [16], and for patients with chronic renal insufficiency (CRI), 2% and 2.3% per year in Korea [17] and Taiwan [9], respectively. More recently, the incidence of SCARs between 2008 and 2015 in Taiwan has been estimated at 0.10% per year; older estimations based on 2001–2004 data were close to 0.30% per year [18]. With this heterogeneity between and within populations, a search for genetic and clinical risk factors is needed.

Several strong risk factors for allopurinol-induced SCARs include recent (< 3 months) allopurinol introduction, high allopurinol starting dose, impaired renal function, and HLA-B*58:01 positivity [19–22]. The link between SCARs and the HLA-B*58:01 allele was first identified in Han Chinese in Taiwan [23]. The strength of the association was reported to vary across ethnicities. Strong associations were found in Han Chinese, Korean, and Thai people [24–26], whereas studies of Japanese and Caucasian populations reported lower allele prevalence and weaker associations [27–29]. Variations in the frequency of the at-risk allele across ethnicities appeared to explain at least in part the racial variations in the incidence of SCARs [12, 13]. To reduce the incidence of SCARs, routine HLA-B*58:01 screening in candidates for allopurinol was found cost-effective and recommended for certain ethnicities with high allele prevalence and a strong association [1, 16, 18, 30, 31]. In contrast to SCARs, for allopurinol-induced MCARs, risk factors have been little investigated.

Regarding its high therapeutic effectiveness and affordable cost, allopurinol is commonly prescribed to treat gouty arthritis and hyperuricemia in both public healthcare centers and private clinics in Vietnam. Kinh Vietnamese, the major ethnic group in Vietnam, present genomic profile and HLA allele distribution similar to surrounding populations including Thai and Han Chinese [32, 33]. However, little is known about allopurinol-induced skin reactions in Kinh Vietnamese population, particularly the clinical risk factors and the association with HLA-B*58:01. The aim of this study was to investigate the risk factors, including HLA-B*58:01 carriage, for MCARs and SCARs in Kinh Vietnamese, who account for up to 90% of the nearly 100 million inhabitants in Vietnam.

## Methods

### Patient recruitment

The study was approved by the Ethics Committee of University of Medicine and Pharmacy at Ho Chi Minh City (HCMC) (HEC/IRB no. 198/ĐHYD-HĐ). Oral and written informed consent was obtained for all patients. All patients were self-identified as Kinh Vietnamese and were prospectively recruited in HCMC. Patients diagnosed with allopurinol-induced SCARs were prospectively recruited in hospital departments (mostly Tropical Diseases, Cho Ray Hospital), where they had been admitted because of the severity of their skin reaction. Patients with mild skin reactions were recruited at the Vien Gut Clinic and at the outpatient clinic of the same hospitals. Patients who had been taking allopurinol and did not show any skin reactions 3 months after the latest escalated dose were prospectively and consecutively recruited at the Vien Gut Clinic as allopurinol-tolerant controls.

### Clinical diagnosis and data collection

Physicians in charge diagnosed the skin reactions to allopurinol. Allopurinol had to be used for < 90 consecutive days, and patients with concomitant use of another drug with high allergy potential were excluded. Photographs of skin manifestations were reviewed and confirmed by certified dermatologists. SCARs were diagnosed according to RegiSCAR criteria [34]. Patients with a combination of high fever, maculopapular rash, lymph node enlargement, blood count abnormalities, and internal organ involvement were classified as having DRESS syndrome. Liver and kidney involvements were defined as more than a two-fold increase of serum liver transaminase levels from the upper limit of the reference range and a 1.5-fold increase of serum creatinine level from the pre-eruption value, respectively. Unspecific skin eruptions not classified as any type of SCAR were considered MCARs.

Clinical history, laboratory test results, and treatment regimen were documented. Concomitant diuretics intake was defined as thiazide or loop diuretics taken continuously during the allopurinol use. A baseline (before skin reaction) estimated glomerular filtration rate (eGFR) < 60 mL/min/1.73 m^2^ was considered CRI.

### DNA extraction and HLA-B*58:01 genotyping

For each patient, 2 ml peripheral venous blood was collected in a tube coated with ethylenediaminetetraacetic acid, and genomic DNA of mononuclear blood cells was extracted by using the GeneJET Whole Blood Genomic DNA purification mini kit (Thermo Fisher Scientific, Waltham, MA, USA). The DNA concentration was measured by using the NanoDrop 2000 Spectrophotometer.

The presence of HLA-B*58:01 allele was first determined by real-time polymerase chain reaction (PCR) sequence-specific amplification with the PG5801 Detection Kit (Pharmigene, Taipei). All PCR reactions were performed with Mastercycler Realplex4 Epgradient S (Eppendorf, Hamburg, Germany). The zygosity of HLA-B*58:01-positive samples was identified by DNA sequencing. In brief, PCR for HLA-B exon 2, 3 and the surrounding region was performed with 5-UT-F and Bin3M13-R primers (Supplementary table 1). Amplification involved using Mastercycler@proS (Eppendorf, Hamburg, Germany) with the following conditions: one cycle at 98 °C for 3 min and 40 cycles of denaturation at 98 °C for 20 s, annealing at 62 °C for 20 s, extension at 72 °C for 1 min, and a final extension at 72 °C for 2 min. The length of amplified products was 1073 bp and was confirmed by electrophoresis on 1% agarose gels with Diamond Nucleic Acid Dye (Promega, Madison, WI, USA). PCR products were cleaned with Exosap-IT glycerol solution (Thermo Fisher Scientific) and sequenced by using the BigDye Terminator v3.1 Cycle Sequencing kit (Applied Biosystems, Foster City, CA, USA) with Seq-BIn2-R and Bin3M13-R primers (Supplementary Table 1). Sequencing reactions were analyzed with the use of ABI 3130 Genetic Analyzer (Applied Biosystems, Foster City, CA, USA). Results were compared with the reference sequence of HLA-B*58:01 from the IMGT database [35]. Only samples that 100% matched the reference sequence and with unambiguity were considered homozygous. In the case of ambiguity, the results were identified as heterozygous.

All techniques were performed in the Center for Molecular Biomedicine of the University of Medicine and Pharmacy at Ho Chi Minh City according to the manufacturer’s protocol.

### Statistical analysis

Descriptive data are presented as mean (standard deviation, SD) for quantitative variables or number (percentage, %) for qualitative variables. Differences were first tested between the 3 groups, then between controls and SCAR and MACR groups for items with a global significant difference. Global differences were tested by Kruskal-Wallis rank-sum test and Fisher exact test for quantitative and categorical variables, respectively. Two-by-two group comparisons were performed with the Wilcoxon rank-sum and Fisher exact tests for quantitative and categorical variables, respectively. The strength of the association was estimated by odds ratios (ORs) with 95% confidence intervals (95% CIs). Considering that some contingency tables include null counts, Firth’s bias-reduced penalized-likelihood logistic regression was systematically applied to obtain valid OR estimates [36, 37]. Due to the small number of cases, we cannot adjust on all potential confounders without being at risk of model over-parametrization. Thus, we proposed to stratify the impact of HLA-B*5801 on eGR, diuretic intake, and age above 65 years. Sensitivity and specificity of HLA-B*58:01 allele presence and positive and negative likelihood ratios with 95% CIs were estimated. Predictive and negative predictive values (PPV and NPV) depend on the incidence of SCARs in the studied population [38] and were estimated from previously estimated incidences of SCARs in Asian populations. All tests were two-sided, with *P* < 0.05 considered statistically significant. All statistical analyses were performed with R v3.6.1 [39].

## Results

In total, 105 patients with allopurinol-induced skin reactions were recruited from October 2017 to May 31, 2018; 74 had MCARs, which developed after a mean allopurinol exposure of 18.5 days (range 1 to 89 days); one additional patient was excluded because he took celecoxib and omeprazole in addition to allopurinol when he developed his skin reaction. Thirty-one patients were hospitalized for SCARs (29 SJS/TEN and 2 DRESS), which developed after a mean exposure of 15.4 days (range 1 to 40 days). None of the patients died. We included 395 tolerant controls from October 2017 to May 31, 2018.

### Study population characteristics

The mean (SD) age of the SCAR group was 60 (15.9) years, significantly higher than both the MCAR and control groups (45.7 [11.7] and 45.4 [10.3] years, respectively). All MCAR and control patients were male, but 9 of 31 SCAR patients were female. The control group had the highest mean allopurinol dose at the time of DNA sampling. Other factors, including history of allopurinol-induced skin reaction, asymptomatic hyperuricemia, and concomitant diuretic intake, were more commonly found in the SCAR than MCAR group and were totally absent in controls (Table 1). The proportion of patients with a starting dose of allopurinol > 150 mg/day was higher in the SCAR than MCAR and control groups (87.1% vs 4.1% and 1.8%). Baseline CRI (eGFR < 60 ml/min/1.73 m^2^) was noted in 66.7%, 8.1%, and 1.8% of SCAR, MCAR, and control groups, respectively. Of note, serum creatinine had not been measured before the toxic reaction in 13 SCAR patients. Overall, 29 (93.6%) of all SCAR patients, including 27 of the 29 SJS/TEN patients and the 2 DRESS patients, were HLA-B*58:01 positive, whereas only a few MCAR patients (6/74, 8.1%) and controls (29/395, 7.3%) carried the allele (Table 1). The proportion of HLA-B*58:01-positive patients who were homozygote for the allele was similar in the SCAR, MCAR, and control groups (25.9%, 16.4%, and 24.1%, respectively).

**Table 1:** Characteristics of Vietnamese Kinh people with cutaneous adverse reactions (MCARs), rare severe CARs (SCARs), and controls (no reaction to allopurinol)

	SCARs (*n* = 31)	MCARs (*n* = 74)	Controls (*n* = 395)	***P*** value
**Demographic features**				
Age, years, mean (SD)	60.0 (15.9)	45.7 (11.7)	45.4 (10.3)	< 0.0001
≤ 40	2 (6.9)	29 (39.2)	135 (34.2)	
40–65	16 (55.2)	42 (56.8)	249 (63.0)	
> 65	11 (37.9)	3 (4.0)	11 (2.8)	
Female, *n* (%)	9 (29.0)	0	0	< 0.0001
**History of allopurinol-induced skin reaction**, *n* (%)	2/27 (7.4)	2 (2.7)	0	0.003
**Indication for allopurinol,*n*(%)**				
Gout	14 (45.2)	72 (97.3)	395 (100)	< 0.0001
Asymptomatic hyperuricemia	17 (54.8)	2 (2.7)	0	< 0.0001
**Allopurinol intake**				
Daily dose at reaction onset, mg, mean (SD)	303.4 (97.22)	243.2 (110.2)	369.1 (103.8)	< 0.0001
Daily starting dose, mg/day, mean (SD)	303.2 (84.6)	156.1 (29.8)	152.7 (19.8)	<0.0001
Daily starting dose > 150 mg, *n* (%)	27 (87.1)	3 (4.1)	7 (1.8)	
**Concomitant diuretics intake,*n*(%)**	7/26 (26.9)	1 (1.4)	0	< 0.0001
**Comorbidities,*n*(%)**				
Hypertension	8/15 (53.3)	4 (5.4)	99 (25.1)	< 0.0001
Type 2 diabetes	6/15 (40.0)	3 (4.1)	24 (6.1)	0.0003
Coronary heart disease	1/15 (6.7)	0	9 (2.3)	0.16
Dyslipidemia	6/13 (46.2)	6 (8.1)	69 (17.5)	0.003
eGFR < 60 ml/min/1.73 m^2^	12/18 (66.7)	6 (8.1)	7 (1.8)	
**HLA typing,*n*(%)**				
HLA-B*58:01-positive	29 (93.5)	6 (8.1)	29 (7.3)	
HLA-B*58:01 homozygote	7/27 (25.9)	1 (16.4)	7 (24.1)	

*eGFR* estimated glomerular filtration rate

### Identified risk factors for allopurinol-induced MCARs

For allopurinol-induced MCARs, we found only three risk factors: CRI, history of allopurinol-induced skin reaction, and asymptomatic hyperuricemia (Table 2). HLA-B*58:01 positivity was not associated with MCARs.

**Table 2:** Identified risk factors for allopurinol-induced MCARs

Risk factors	MCAR (*n* = 74)	Control (*n* = 395)	OR (95% CI)	***P*** value
Age, years, mean (SD)				
≤ 40	29 (39.2)	135 (34.2)	1.28 (0.76–2.13)	0.35
40–65	42 (56.8)	249 (63.0)	1	
> 65	3 (4.0)	11 (2.8)	1.79 (0.44–5.67)	0.38
Female, *n* (%)	0	0		1
History of allopurinol-induced skin reaction, *n* (%)	2 (2.7)	0	27 (2–3777)	0.01
Asymptomatic hyperuricemia, *n* (%)	2 (2.7)	0	NA	NA
Allopurinol starting dose > 150 mg/day, *n* (%)	3 (4.1)	7 (1.8)	2.5 (0.6–8.7)	0.19
Concomitant diuretics intake, *n* (%)	1 (1.4)	0	16 (0.9–2365)	0.06
eGFR < 60 ml/min/1.73 m^2^, *n* (%)	6 (8.1)	7 (1.8)	4.9 (1.6–14.6)	0.006
HLA-B*58:01-positive, *n* (%)	6 (8.1)	29 (7.3)	1.18 (0.45–2.7)	0.72

*OR* odds ratio, *95% CI* 95% confidence interval, *NA* not applicable

### Identified risk factors for allopurinol-induced SCARs

**Fig. 1:**
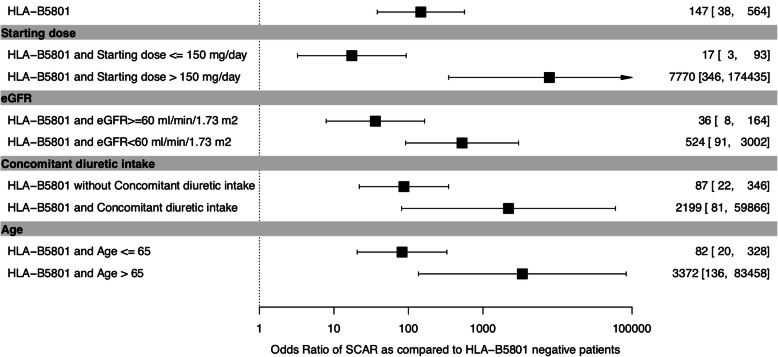
Probability of severe cutaneous adverse reactions (SCARs) in the total population of Vietnamese Kinh people and in those with starting allopurinol dose > 150 mg/day, renal failure, concomitant diuretic intake, and age ≥ 65 years. Data are odds ratios (95% confidence intervals)

We identified seven risk factors for allopurinol-induced SCARs, with significant difference and high ORs between SCAR and control groups: female sex, age > 65 years, history of allopurinol-induced skin reaction, high starting dose of allopurinol, concomitant diuretics intake, eGFR < 60 ml/min/1.73 m^2^, and HLA-B*58:01 positivity (Table 3). More than half of the SCAR patients received allopurinol to treat asymptomatic hyperuricemia in contrast to our controls who all had gout, as the Vien Gut Medical Centre, where controls were recruited, follows the international guideline not to treat asymptomatic hyperuricemia. SCAR patients also more frequently had hypertension (*P* = 0.03) and dyslipidemia (*P* = 0.02) than controls. Mean allopurinol starting dose and ratio of starting dose to eGFR were higher with SCARs. An allopurinol starting dose > 150 mg/day was strongly associated with SCARs. SCARs developed only within patients with a ratio of starting dose to eGFR > 2.74. A similar strong association was found using a threshold ratio of 4 (corresponding to the first quartile of the starting dose/eGFR ratio in SCAR patients) with the advantage of greater specificity. The probability of HLA-B*58:01 positivity with the occurrence of allopurinol-induced SCARs was high (OR 147 [95% CI 38–564]) and was stronger with the concomitant risk factors age > 65 years (3372 [136–83,458]), eGFR < 60 ml/min/1.73 m^2^ (524 [91–3002]), concomitant diuretics intake (2199 [81–59,866]), and starting dose > 150 mg/day (7770 [346–174,435]) as compared with HLA-B*58:01 negativity (Fig. 1). Of note, all patients with SCARs and with renal insufficiency or > 65 years old were HLA-B*58:01 positive.

**Table 3:** Identified risk factors for allopurinol-induced SCARs

Risk factors	SCARs (*n* = 31)	Control (*n* = 395)	OR (95% CI)	***P*** value
Age, years, mean (SD)				
≤ 40	2 (6.9)	135 (34.2)	0.28 (0.05–0.91)	0.04
40–65	16 (55.2)	249 (63.0)	1	
> 65	11 (37.9)	11 (2.8)	15.1 (5.8–40.1)	< 0.0001
Female, *n* (%)	9 (29.0)	0	333 (40–43,453)	< 0.0001
History of allopurinol-induced skin reaction, *n* (%)	2/27 (7.4)	0	78 (6–10,808)	0.004
Asymptomatic hyperuricemia, *n* (%)	15/27 (55.6)	0	NA	NA
Allopurinol starting dose > 150 mg/day, *n* (%)	27 (87.1)	7 (1.8)	316 (101–1224)	< 0.0001
Concomitant diuretics intake, *n* (%)	7/26 (26.9)	0	304 (35–40,018)	< 0.0001
eGFR < 60 ml/min/1.73 m^2^, *n* (%)	12/18 (66.7)	7 (1.8)	100 (32–353)	< 0.0001
HLA-B*58:01-positive, *n* (%)	29 (93.5)	29 (7.3)	147 (45–746)	< 0.0001

*NA* not applicable

The interest of HLA-B*58:01 testing to predict SCARs in Kinh Vietnamese was determined by several approaches. Sensitivity and specificity were estimated at 93.5% (95% CI 78.6–99.2) and 92.5% (89.8–94.7), respectively. Positive and negative likelihood ratios were estimated at 12.5 (9.0–17.5) and 0.07 (0.02–0.27). Therefore, both tests strongly supported the value of HLA-B*58:01 genotyping, because likelihood ratios > 10 and < 0.1 are considered strong evidence to rule in or rule out diagnoses, respectively [40]. Considering incidence figures observed in South Asia populations (see the “Introduction” section), a SCAR incidence < 0.30% per year led to estimate a PPV < 3.6% and NPV > 99.9%, respectively. With retained prevalence < 1/1000, the estimated PPV decreased to 1.2% and the NPV remained > 99.9%. In our patients with age > 65 years or eGFR < 60 ml/min/1.73 m^2^ in whom the SCAR prevalence could increase to 2/100, estimated PPVs reached 22.2% and 12.2%, respectively, and NPVs were still > 99.9%.

## Discussion

The most common adverse reactions to allopurinol are MCARs [11, 41], which, in our study, associated with CRI, previous skin reaction to allopurinol, and management of asymptomatic hyperuricemia. Only 8.1% of MCAR patients were positive for HLA-B*58:01, for an insignificant strength of association. This finding agrees with reports from Portugal, Australia, and Korea [17, 29, 41]. In contrast, studies of Han Chinese and Thai people with MCARs revealed high HLA-B*58:01 carriage (85–100%) and a strong association similar to that of SCARs [24, 42].

SCARs are extremely severe and life-threatening and are the most important hazard for patients with allopurinol treatment. Many studies have focused on the identification of risk factors to minimize the risk of SCARs [3, 21, 23, 25, 29, 43], but these have been little investigated in Vietnamese people. Our study found several non-genetic and genetic risk factors for allopurinol-induced SCARs in the Kinh Vietnamese population, which confirmed the findings for other ethnicities [3, 10, 22].

We found that starting allopurinol at > 150 mg/day was highly associated with SCARs (OR 316 [95% CI 101–1224]), which confirmed previous results about the importance of starting dose [4, 13, 21], even though the starting dose in our tolerant controls was higher than the dose of ≤ 100 mg/day usually recommended [1, 44]. Our tolerant controls were recruited at the Vien Gut Medical Centre, where the policy is to restrict allopurinol prescription to patients with eGFR > 60 ml/min/1.73 m^2^ and to start the drug at 150 mg/day (owing to the sole availability of 300 mg tablets in this center), with subsequent escalation of the dose by 150 mg/day every month until the desirable uricemia target is reached. Because no SCAR was observed in more than 10,000 gouty patients prescribed allopurinol according to this protocol at the Vien Gut Medical Centre over the last 4 years, 150 mg/day may be an acceptable starting dose in Vietnamese patients without renal insufficiency. This approach is supported by the fact that all the SCAR patients in the present study had a starting dose of at least 300 mg/day or eGFR < 60 ml/min/1.73 m^2^. Allopurinol was finally given at a significantly higher dose in our tolerant controls than in those with skin reactions, which confirms the involvement of starting dose and not maintenance dose in provoking skin reactions. Strikingly, more than 50% of patients with SCARs had received allopurinol for asymptomatic hyperuricemia. Other investigators also found that allopurinol prescription for asymptomatic hyperuricemia notably increased the risk of SCARs [4]. Thus, great caution is warranted in treating asymptomatic hyperuricemia with allopurinol.

Concomitant use of diuretics was also a risk factor for allopurinol-induced SCARs (OR 304 [95% CI 35–40,018) similar to other reports [13, 19, 20, 22]. This finding could be related to the decreased renal clearance of oxypurinol, which has been documented in patients taking thiazides [45] and furosemide [46]. However, the exact role of diuretics in allopurinol-induced skin reactions remains controversial, because studies of both Asians [4, 17, 23] and Caucasians [5] showed that taking diuretics with allopurinol, after adjustment for other factors, in particular comorbidities, did not significantly increase the risk of hypersensitivity. We did not use multiple adjustments in our study because of the limited number of included SCAR patients. Of note, our 7 SCAR patients who took diuretics took them for hypertension.

CRI has been highlighted as an independent risk factor for allopurinol-induced SCARs in many studies [13, 20, 22, 24, 43, 47], leading to recommend limiting the allopurinol dose based on baseline eGFR to minimize the risk [44]. Most regulatory agencies declared this recommendation, but it has recently been debated because it appeared to compromise the urate-lowering effect of allopurinol in renal patients [48]. Also, in some studies, SCARs seemed to occur regardless of adjustment of allopurinol dose to renal function [3, 5, 23, 48], whereas in others, plasma oxypurinol level was correlated with prognosis rather than SCARs [10, 20, 48]. In our study, CRI was associated with both SCARs (OR 100 [95% CI 32–353]) and MCARs (OR 4.9 [1.6–14.6]). However, this result must be taken with caution because 13 of our 31 SCAR patients had no creatinine measurement before their eruption, and patients with renal failure were not included in our tolerant control group because of the mode of recruitment.

Several in vitro studies shed some light on the idiosyncratic characteristics of allopurinol-induced SCARs. Delayed hypersensitivity via CD8+ T cells seems to play a central role in the pathogenesis of SCARs, involving the antigen presentation process. The response requires a sufficient amount of drug molecules binding to peptide–major histocompatibility complex and also a certain specificity between antigen-presenting cells and CD8+ T cells [49]. This finding led to two crucial categories of risk factors: the first includes drug dose, reduced clearance due to impaired renal function or diuretics, and increased oxypurinol plasma concentration, and the second, regulation of oxypurinol presentation to immune cells by immune receptors such as HLA-B*58:01 [49].

HLA-B*58:01 has emerged as a promising genetic marker to predict allopurinol-induced SCARs. However, substantial difference by ethnicity was an important determinant of clinical cost-effective analyses: HLA-B*58:01 screening was beneficial in certain countries with high-risk ethnic groups but was ineffective in others [50]. Our Ho Chi Minh study found a strong association between HLA-B*58:01 and allopurinol-induced SCARs (OR 147 [95% CI 45–745]) in accordance with a previous study in Hanoi [51]. Also, the frequency of the HLA-B*58:01 genotype was 7.3% in our tolerant gouty patients, similar to the results for the general Kinh population of both South and North Vietnam [33, 52]. Hence, our results suggest that Kinh Vietnamese are another high-risk group in whom HLA-B*58:01 would be worth genotyping before prescribing allopurinol.

Within the HLA-B locus, the number of 58:01 alleles can be one (heterozygous) or two (homozygous) and can result in a gene-dosage effect. Ng et al. reported a higher incidence of SCARs in homozygotes than heterozygotes (OR 72.45 vs 15.25) [47]. Shim et al. showed similar results, but the association was not statistically significant [53]. In our study, risk of SCARs was not higher in patients homozygous than heterozygous for HLA-B*58:01.

The PPVs and NPVs estimated in our work agree with previous reports finding a PPV for carriage of < 10% [47, 54], whereas with a non-carriage state, the probability of allopurinol-induced SCAR was extremely unlikely: the NPV was almost 100% [55]. Our results for patients with renal failure also agree with those of Jung et al., who found a PPV and NPV of 18% and 100%, respectively, in a Korean population with CRI [17].

Given both the cost-effectiveness of allopurinol therapy and the rare occurrence of SCARs, a single risk factor could be insufficient to justify absolute restraint from this drug. To date, the most widely recommended approach to risk evaluation of allopurinol-induced SCAR has been to combine multiple well-studied factors. Lee et al. proposed screening for HLA-B*58:01 in patients with one or more characteristics including baseline eGFR < 60 mL/min/1.73 m^2^, co-administration with multiple drugs especially diuretics, East Asian or Southeast Asian background, and history of allopurinol intolerance [41]. Jung et al. reported a 6-fold rate of SCARs in patients with HLA-B*58:01 carriage and CRI versus those with normal renal function (18% vs 2.7%) [17], which resulted in necessary avoidance of allopurinol in CRI patients carrying the at-risk allele. Ng et al. reported that a combination of HLA-B*58:01 and poor renal function significantly improved the ORs, PPV, and area under the receiver operating characteristic curve as compared with a single risk factor [47].

**Fig. 2:**
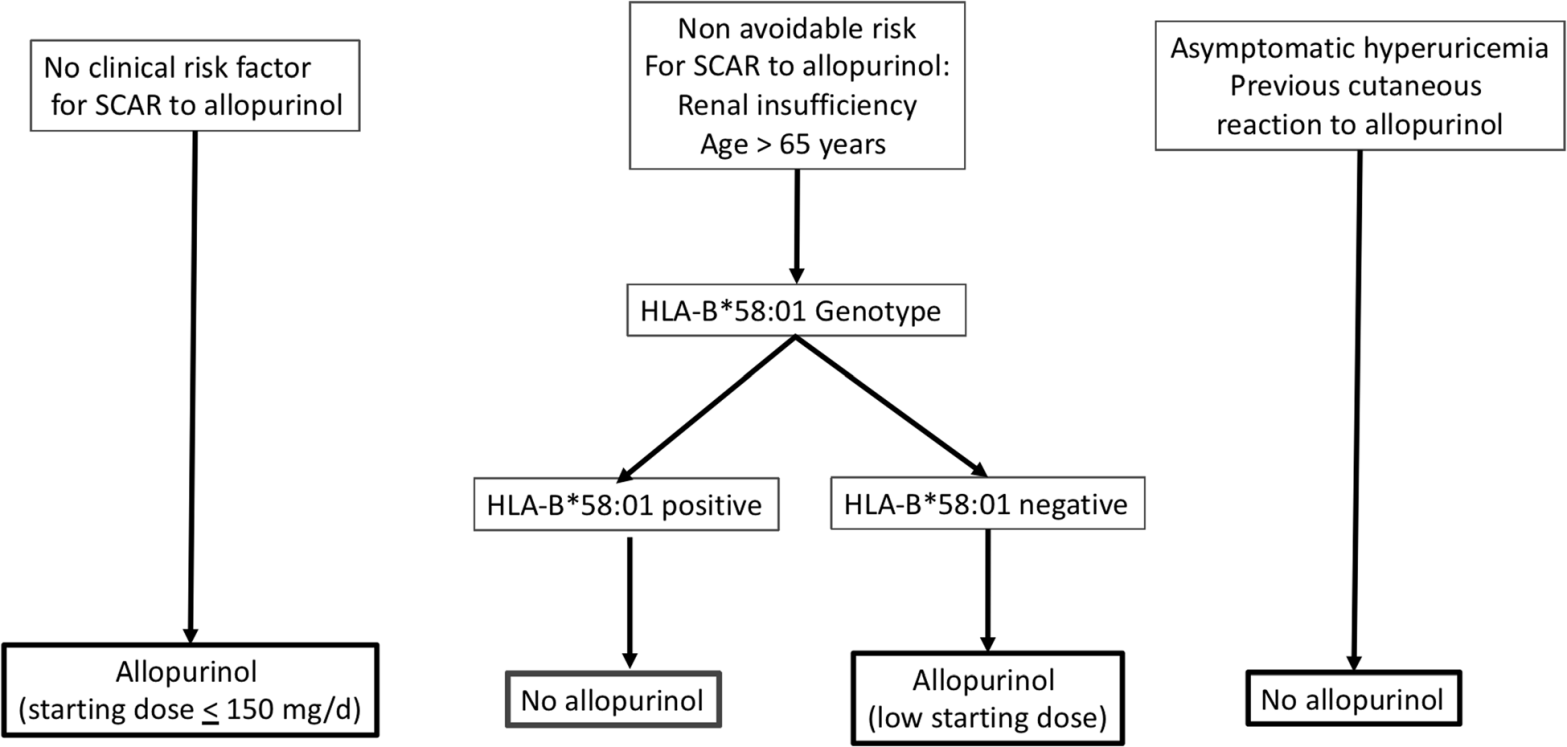
A proposed decision tree for allopurinol prescription in Kinh Vietnamese

In our study of Kinh Vietnamese, with several risk factors (8 for SCARs and 3 for MCARs), a number should be avoided such as starting allopurinol at a high dose and treating asymptomatic hyperuricemia or a history of skin intolerance. Our results support that patients with no clinical risk factor could be started on allopurinol, with a starting dose not exceeding 150 mg/day. HLA typing could be advised in patients taking diuretics, older people, and those with CRI, to guide allopurinol prescription, which could be allowed in HLAB*58:01-negative patients, at a low starting dose (Fig. 2), as supported by the high ORs for SCARs associated with the at-risk allele in these patients (Fig. 1).

This study has limitations. Despite prospective enrolment, a number of data were missing, especially in the SCAR patient group as these patients went to the hospital on emergency and had not been followed-up as out-patients. The mode of recruitment of tolerant controls at the Vien Gut Medical Centre excluded asymptomatic hyperuricemia patients as this center follows the policy not to treat asymptomatic hyperuricemia. This made a valid comparison with cutaneous intolerance patients impossible. The case-control study design we used exposes to a higher risk of bias than a cohort study. The main risk of bias lies in the difficulty of collecting the exposure factor (recall bias), but for a genetic study, this risk is very low as exposure is assessed by PCR. Moreover, in the presence of rare disease or event, it is the only way to achieve results within a reasonable time and at a reasonable cost. Nevertheless, we cannot rule out that confounders such as advanced age or comorbidities could impact the results of our study. Due to the small number of cases, we could not adjust on all potential confounders without being at risk of model over-parametrization. Thus, we proposed to stratify the impact of HLA-B*5801 on eGFR, diuretic intake, and age above 65 years (Fig. 1). Results still showed an impact of the HLA-B*5801 and seem robust when age or renal insufficiency are taking into account. In logistic regression analysis of small or sparse data sets, results obtained by classical maximum likelihood methods cannot be generally trusted. This situation has been termed “separation,” and it typically occurs whenever no or few events are observed in one of the two groups defined by a dichotomous covariate and in the presence of highly predictive risk factors [37, 56]. In such situation, Heinze and Schemper have demonstrated that Firth’s modification of the logistic model unbiased estimates and valid confidence intervals. However, the width of confidence intervals should lead to relativize the value of some point estimates.

## Conclusion

The multiple risk factors for allopurinol-induced SCARs in Kinh Vietnamese identified in this study should further raise awareness about the risk of hypersensitivity and the need for careful evaluation before allopurinol prescription. Considering that the cost of HLA*58:01 testing is gradually decreasing in Vietnam, identifying genetic risk factors could be extremely valuable to rule out candidates with inevitable risk factors for allopurinol treatment. As a result, a urate-lowering strategy could be wisely tailored to achieve both therapeutic safety and effectiveness.

## Supplementary information

**Additional file 1: Table S1.** Primers used for PCR and sequencing HLA-B.

## Data Availability

The datasets used or analyzed during the current study are available from the corresponding author on reasonable request.

## Abbreviations

CAR: Cutaneous adverse reaction
CI: Confidence interval
CRI: Chronic renal insufficiency
DRESS: Drug reaction with eosinophilia and systemic symptoms
eGFR: Estimated glomerular filtration rate
HCMC: Ho Chi Minh City
HLA: Human leucocyte antigen
MCAR: Mild cutaneous adverse reaction
NA: Not applicable
NPV: Negative predictive value
OR: Odds ratio
PCR: Polymerase chain reaction
PPV: Positive predictive value
SCAR: Severe cutaneous adverse reaction
SD: Standard deviation
SJS/TEN: Stevens-Johnson syndrome/toxic epidermal necrosis
