# Supplementary material for: Risk factors for cutaneous reactions to allopurinol in Kinh Vietnamese: results from a case-control study
Source: Arthritis Res Ther. 2020 Aug 3;22:182. doi: 10.1186/s13075-020-02273-1 (PMC7397637; doi:10.1186/s13075-020-02273-1)
Supplement: Supplementary file 1 — Additional file 1: Table S1. Primers used for PCR and sequencing HLA-B. [file 13075_2020_2273_MOESM1_ESM.docx]

Supplementary table 1. Primers used for PCR and sequencing HLA-B

| Name | Sequence (5’ to 3’) |
| --- | --- |
| 5UT-F | GACTCAGAATCTCCTCAGACGCCGA |
| Bin3M13-R | GGCCATCCCCGGCGACCTAT |
| Seq-BIn2-R | GGATCTCGGACCCGGAG |
